# Emergency surgery for tubo-ovarian abscess identified extended-spectrum beta-lactamase-producing *Escherichia coli*: the first case presentation revealing causative bacteria

**DOI:** 10.1186/s40792-015-0069-6

**Published:** 2015-08-15

**Authors:** Teppei Tokumaru, Yasuo Shima, Takehiro Okabayashi, Kazutoshi Hayashi, Yorito Yamamoto, Kazuhide Ozaki, Jun Iwata

**Affiliations:** Department of Gastroenterological Surgery, Kochi Health Sciences Center, 2125-1 Ike, Kochi-City, Kochi 781-8555 Japan; Department of Obstetrics and Gynecology, Kochi Health Sciences Center, 2125-1 Ike, Kochi-City, Kochi 781-8555 Japan; Department of Diagnostic Pathology at Kochi Health Science Center, 2125-1 Ike, Kochi-City, Kochi 781-8555 Japan

**Keywords:** Emergency surgery, Tubo-ovarian abscess, Extended-spectrum beta-lactamase, Surgery

## Abstract

We report herein a 41-year-old female with a tubo-ovarian abscess (TOA), which microbial cultures showed to contain extended-spectrum beta-lactamase (ESBL)-producing *E. coli,* a causative agent of community-acquired infection. The patient initially presented with acute abdominal pain and back pain. Pelvic computed tomography and transvaginal ultrasonography revealed multiple cystic lesions in the bilateral ovaries that suggested TOA. An emergency laparotomy was therefore performed due to the potential for life-threatening septic shock from the TOA-associated pelvic inflammatory disease. Microbial cultures of postoperative fluid discharge from the placed intra-abdominal catheter, vaginal secretions, urine, blood, and feces detected ESBL-producing *E.coli.* In summary, we successfully performed emergency surgery for life-threatening septic TOA caused by ESBL-producing *E. coli* infection.

## Background

A tubo-ovarian abscess (TOA) can develop in reproductive age women with pelvic inflammatory disease (PID). The cause of TOA is mostly ascending infection through the uterus (mainly sexually transmitted diseases) due to *Neisseria gonorrhoeae*, *Chlamydia*, *Escherichia coli* (*E. coli*), and/or endogenous bacteria of the vagina and cervix [1, 2]. The management of TOA is a fundamentally conservative treatment with systemic broad-spectrum antibiotics. However, a TOA can have serious and potentially life-threatening consequences when there is a risk of abscess rupture. In such cases, antibiotic therapy is not sufficient for treating the TOA, and surgical drainage must be performed [3–6].

In this case report of a patient with TOA, surgery was indicated after the initial diagnosis due to the immediate threat of clinically dangerous septic shock. Although the abscesses were not ruptured, infections to the vagina, uterus, and bilateral ovaries by extended-spectrum beta-lactamase (ESBL)-producing *E. coli* had the potential to induce septic shock and disseminated intravascular coagulation, both of which can be life-threatening conditions. ESBL-producing bacteria infect individuals with impaired immunity, have resistance to many antibiotics, and can cause nosocomial infection [7–12]. To our knowledge, this is the first reported case of a patient with TOA caused by ESBL-producing *E. coli*. and treated with emergency surgery.

## Case presentation

A 41-year-old woman presenting with acute abdominal and back pain was admitted into a neighboring hospital. She was initially diagnosed with a urinary tract infection and received systemic antibiotic therapy for 2 days. She complained of worsening abdominal pain and high fever, suggesting PID due to bacterial infection into the bilateral ovaries, and was subsequently referred to our hospital. She was previously healthy with no history of heavy alcohol consumption, smoking, diabetes mellitus, or overseas travel.

**Fig. 1:**
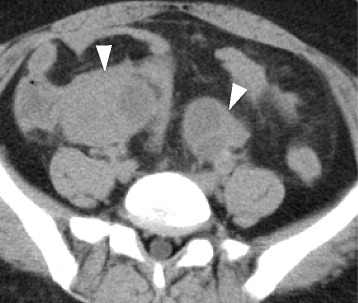
Abdominal computed tomography revealed thick-walled multilocular cystic lesions at the bilateral ovaries (*white arrowheads*)

**Fig. 2:**
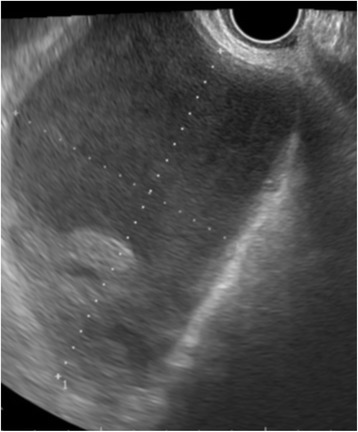
Transvaginal sonography revealed cystic lesions with septum at the bilateral ovary

Physical examination revealed a body temperature of 40 degrees, mild abdominal pain, and markedly anemic conjunctiva. Her abdomen was soft, but showed mild tenderness and rebound pain. Hematological and biochemical testing showed a red blood cell count of 280 × 10^4^/uL (normal range, 370–490 × 10^4^/uL), hematocrit of 22.5 % (normal range, 34–49 %), hemoglobin (Hb) level of 7.3 g/dL (normal range, 11.2–15.7 g/dL), white blood cell count of 16,500/uL, platelet count of 18,000/uL, C-reactive protein level of 18.0 mg/dL, aspartate transaminase (AST) of 146 IU/L (normal range, 8–38 IU/L), alanine transaminase (ALT) of 79 IU/L (normal range, 4–44 IU/L), alkaline phosphatase (ALP) of 563 IU/L (normal range, 115–359 IU/L), lactate dehydrogenase (LD) of 899 IU/L (normal range, 106–211 IU/L); total protein concentration of 4.6 g/dL (normal range, 6.7–8.3 g/dL), albumin levels of 2.0 g/dL (normal range, 3.8–5.3 g/dL), cholinesterase concentration of 104 IU/L (normal range, 229–521 IU/L), creatinine concentration of 3.1 mg/dL (normal range, 0.4–0.8 mg/dL), prothrombin time of 44.1 % (normal range, 77.0–104.0 %), activated partial thromboplastin time, 47.7 s (normal range, 20.0 to 40.0 s), fibrin/fibrinogen degradation products at 239.4 ug/mL (normal range, 0.0–10.0 ug/mL), D-dimer levels of 75.1 ug/mL (normal range, 0.0–1.0 ug/mL), antithrombin 3 activity of 53 % (normal range, 81–123 %), base excesses of −13.0 mmol/L (normal range, −3.0–3.0 mmol/L), and lactate concentration of 12.9 mmol/L (normal range, 0.5–2.0 mmol/L). Other laboratory tests, including tumor markers (carcinoembryonic antigen, carbohydrate 19–9, and carbohydrate antigen 125) and viral status for hepatitis B status and C status, were normal. Abdominal computed tomography (CT) revealed thick-walled multilocular cystic lesions in the bilateral ovaries, and no ascites in the pelvis (Fig. 1). TOA was strongly suspected, and the patient underwent transvaginal ultrasonography, which demonstrated an 11.1 × 7.9 cm left ovarian cystic mass with low echogenicity, and a 6.3 × 4.4 cm right ovarian mass with a similar echo-texture (Fig. 2). For further definitive diagnosis, ultrasound-guided puncture was conducted in the cystic mass and revealed a purulent content. Together, the clinical presentation and imaging findings provided a diagnosis of TOA. An emergency laparotomy was performed based on the clinical diagnosis of septic shock and already completed DIC due to the TOA.

**Fig. 3:**
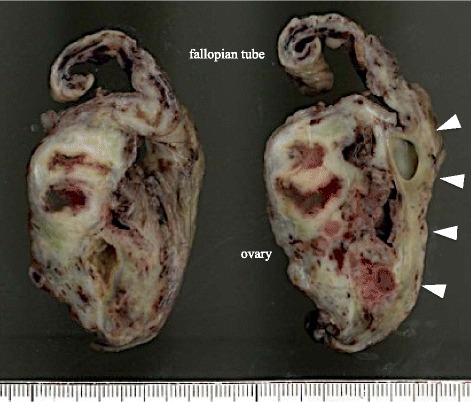
Resected specimen of ovary, showing marked swelling of the bilateral ovaries with intraluminal abscess. Pathological examination revealed the presence of ectopic endometriosis around the ovary (*white arrowheads*)

**Fig. 4:**
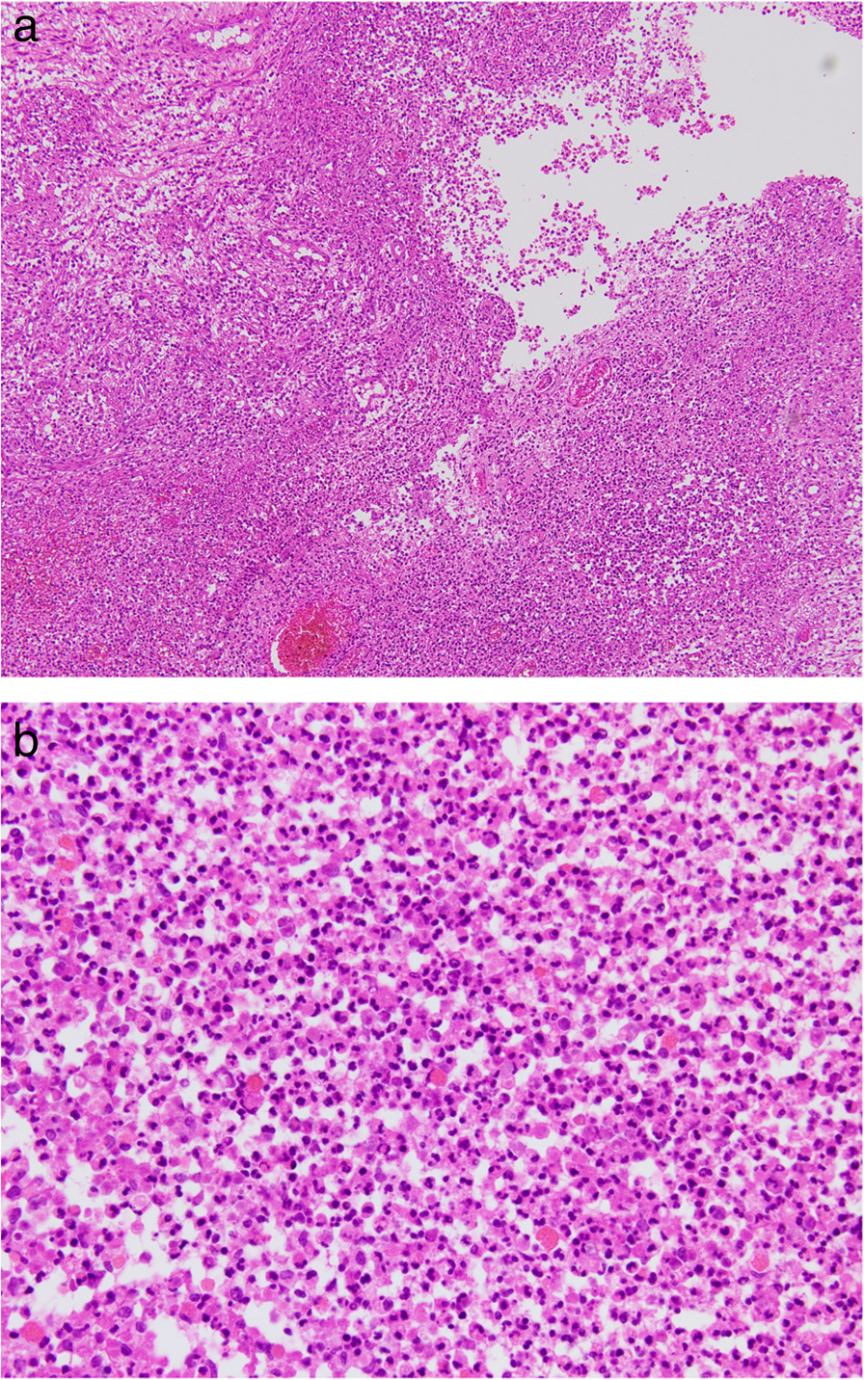
**a** HE stain ×10. The ovary showed a high degree of neutrophil infiltration with abscess formation. **b** HE stain ×100. High-power magnification showed a cluster of neutrophils in the ovary

The gross examination during surgery showed inflammation around the bilateral ovaries that was so marked that it prevented identification of individual organs. The bilateral ovaries and uterus were firm to palpate; however, the vagina in the pelvic cavity was relatively soft on palpitation. The inflammatory lesions including the ovaries and the uterus containing a uterine fibroid were resected to control oozing hemorrhage from the pelvic cavity because the patient showed evidence of DIC. During this procedure, the cystic component was partially damaged and discharged a small amount of purulent fluid, which subsequent cultures showed to contain ESBL-producing *E.coli*. Resected specimens of the bilateral ovaries showed marked swelling and thick-walled multilocular cystic lesions with intraluminal abscesses (Fig. 3). Microscopic examination of the resected specimen revealed infiltration of foamy cells and neutrophils into the abscess cavity (Fig. 4). No cell types showed atypical mitotic images or pleomorphisms.

Postoperative microbial cultures of the fluid discharge from the placed intra-abdominal catheter, vaginal secretions, urine, blood, and feces detected ESBL-producing *E.coli*. The patient suffered further abscess formation due to infection of the postoperative hematoma in the pelvic cavity, and underwent a repeat laparotomy with abdominal drainage for diffuse peritonitis 12 days after the initial surgery. She subsequently suffered intra-abdominal septic complications, and underwent ultrasound-guided percutaneous drainage on the 27th postoperative day. After the drainage, she made a favorable recovery and was discharged from our hospital 72 days after the first emergency surgery.

### Discussion

TOA is most frequently induced by ascending infection through the uterus due to *Neisseria*, *gonorrhoeae*, *Chlamydia*, *E. coli*, or indigenous bacteria of the vagina and cervix, and it usually follows PID [1, 2]. Herein, we present the first reported case of a patient with TOA caused by ESBL-producing *E. coli*.

ESBL-producing bacteria is well known as one of the multiple-antibiotic-resistant bacterium identified by German Knothe in 1983 [13]. Our case had no comorbidity, including diabetes mellitus, respiratory disease, ischemic heart disease, immune-deficiency disease, and had never been hospitalized following birth. Thus, we considered this case to be a community-acquired infection. Most infections due to ESBL-producing bacteria remain hospital-acquired and are even more common among long-term hospitalized and immunocompromised patients [8]. Recently, however, the incidence of community-acquired infection by ESBL-producing bacteria has gradually increased in Western countries, although it remains rare in Japan, and the increasing numbers reported have been attributed to advances in diagnostic techniques and equipments [12, 14, 15]. In the recent years, it has been reported that the incidence of colonization induced by community-acquired ESBL-producing bacteria from urine and/or feces has been increased, even with patients with no comorbidities in Japan as the West. Therefore, the current study suggested that physicians should pay attention to the community-acquired infection of ESBL-producing bacteria which might have been increased in the future [14, 16, 17].

Treatment of TOA is important to avoid complications such as life-threatening abscess rupture and sepsis, and to preserve fertility, so early detection and treatment of TOA can prevent such adverse outcomes. However, the peculiar clinical signs of TOA are absent, such as lower abdominal tenderness, abnormal vaginal or cervical discharge, fever, abnormal vaginal bleeding, dyspareunia, cervical motion tenderness, and adnexal tenderness [18, 19]. Furthermore, it was also very difficult to detect clinically TOA according to laboratory investigations like following; findings, presence of excess leucocytes, and/or C-reactive protein [19]. The differential diagnosis for TOA includes a wide array, such as reproductive system disease, or gastrointestinal inflammation, urinary tract infection. Therefore the diagnosis of TOA based on clinical criteria alone at the early stage is difficult and inaccurate [18, 19]. In the present case, the diagnosis of potential TOA was suggested strongly by subsequent transvaginal ultrasound. Interestingly, this patient with insufficiency of intravenous antibiotics treatment ran a fatal course due to subsequent septic complication for TOA in only two days. In general, ruptured TOA can rapidly become critical for the patient, whereas unruptured TOA commonly progresses slowly [19, 20]. A rapid onset of endotoxin shock and DIC is commonly inferred with Gram-negative *E. coli*, especially in this case with detection of the multiple-antibiotic-resistant ESBL-producing bacteria, which produces endotoxin and generally enter the bloodstream and peritoneal pelvic cavity, causing high fever and acute lower abdominal pain.

Therapeutically, when conservative management with antibiotics is ineffective, surgical management is strongly recommended for TOA [4, 7]. As surgical management, surgical drainage, salpingo-oophorectomy, and abdominal hysterectomy in combination with antibiotics is generally performed in women with TOA [21–23], with the aim of being minimally invasive and as conservative as possible [24–27]. In our case, because the TOA could have developed under septic conditions and thus rapidly induce the critical condition of DIC, we immediately proceeded with surgical treatment by salpingo-oophorectomy and hysterectomy.

In conclusion, we successfully performed emergency surgery for life-threatening septic TOA caused by ESBL-producing *E. coli,* which was not controlled with conservative antibiotic treatment. To ensure the best management of such cases, reporting of cases with similar pathological and bacteriological findings is warranted.

## Conclusions

Aggressive surgery is a valid choice for treating TOA in specific life-threatening situations.

## Consent

Written informed consent was obtained from the patient for publication of this case report and any accompanying images. A copy of the written consent is available for review by the Editor-in-Chief of this journal.
